# Unfolded Protein Response in Glioblastoma: Mechanisms of Proteostasis, Tumor Adaptation, and Therapeutic Relevance

**DOI:** 10.1007/s11064-026-04772-0

**Published:** 2026-05-18

**Authors:** Hasan Onur Caglar

**Affiliations:** Department of Molecular Biology and Genetics, Science Faculty, Erzurum Technical University, Erzurum, 25050 Turkey

**Keywords:** ATF6, Endoplasmic reticulum stress, Glioblastoma, IRE1, PERK, Unfolded protein response

## Abstract

Glioblastoma (GBM) is an aggressive brain tumor that rapidly develops resistance to standard clinical therapies. The tumor microenvironment of GBM is highly hostile, characterized by hypoxia, elevated reactive oxygen species, and severe metabolic stress. These conditions promote protein misfolding, particularly in the endoplasmic reticulum (ER), thereby triggering ER stress. The unfolded protein response (UPR) is an adaptive signaling pathway that mitigates ER stress, restores proteostasis, and promotes cellular survival. Activation of UPR signaling provides a survival advantage to GBM cells under these adverse conditions. This signaling is closely associated with drug resistance and malignant progression in GBM. Furthermore, inhibition of UPR sensors exhibits anticancer effects, highlighting their potential as therapeutic targets in GBM. This review describes the biological functions of UPR sensors and their roles in GBM pathogenesis and treatment response.

## Introduction

Proteins govern a wide range of physiological and cellular processes essential to maintaining homeostasis. Protein function is determined by the acquisition of a correct three-dimensional structure following synthesis, subsequent post-translational modifications, and precise subcellular localization [1]. Impaired protein folding can compromise protein function, thereby disrupting proteostasis and, in turn, cellular homeostasis. Because proteins play essential roles in cellular processes, cells have evolved sophisticated quality-control and stress-response mechanisms to maintain proteostasis [2].

Under diverse stress conditions, perturbations in proteostasis increase the burden of misfolded polypeptides. In response, heat-shock proteins (HSPs) function as molecular chaperones, facilitating the correct folding of nascent chains, promoting refolding of misfolded proteins, or directing irreversibly damaged proteins toward degradation pathways [3]. The persistence of cellular stress determines the capacity of the chaperone system to maintain proteostasis. When the accumulation of misfolded proteins exceeds the chaperone capacity, another adaptation mechanism, the unfolded protein response (UPR), is activated to restore protein homeostasis in the endoplasmic reticulum (ER) [4]. Misfolded proteins accumulate particularly in the ER, where protein synthesis and post-translational modifications occur, triggering this pathway. This adaptive signaling network restores proteostasis by attenuating translation, enhancing chaperone expression, and promoting degradation pathways. Indeed, UPR activation reduces the burden of misfolded proteins through refolding mechanisms and degradation pathways, including ER-associated degradation (ERAD) and autophagy [5]. However, when ER stress is prolonged, the same signaling leads to pro-apoptotic output. Thus, the UPR functions as a central determinant of the balance between cellular survival and death.

Glioblastoma (GBM) tumors are exposed to diverse stressors arising from the tumor microenvironment and therapeutic approaches [6]. These stressors increase the protein-folding burden in GBM cells, leading to the accumulation of misfolded or damaged proteins within the ER. In this context, UPR signaling promotes proteostasis in GBM cells, thereby facilitating cellular adaptation to stress. In GBM, ER stress-induced UPR activation is also associated with drug resistance and tumor progression. Moreover, UPR-targeted approaches have demonstrated anticancer effects in GBM cells, prompting ongoing efforts to therapeutically modulate this pathway [7–9]. This review delineates the biological functions of UPR sensors and examines their mechanistic roles in GBM pathobiology and therapeutic response.

### Unfolded Protein Response

The majority of secreted and membrane proteins are synthesized and folded in the ER, then transported to the Golgi apparatus for further modifications before being delivered to their functional destinations [10]. In this regard, the ER is the most important organelle involved in protein folding and proteostasis. Sensor proteins on the ER membrane initiate the UPR by detecting misfolded proteins in the ER lumen. Cells have highly complex and dynamic systems that enable specific biological responses to particular stimuli. The necessity to respond appropriately to internal and external stimuli is evolutionarily conserved in cells. The presence of misfolded proteins in the ER lumen triggers the UPR to prevent severe cellular disruption [11, 12]. Under basal conditions, ER-resident chaperone GRP78/BiP binds to UPR sensor proteins, including IRE1, PERK, and ATF6, maintaining them in an inactive state. Upon ER stress, GRP78/BiP preferentially binds misfolded proteins, leading to its dissociation from these sensors and thereby initiating UPR signaling [13]. This response mechanism involves the synthesis of chaperone proteins, which regulate protein folding and function. Additionally, it includes mRNA degradation and the suppression of protein synthesis, both of which reduce the accumulation of additional misfolded proteins in the ER lumen [14, 15]. Thus, proper folding of unfolded or misfolded proteins is promoted in the ER lumen, whilst limiting further protein entry into the ER helps prevent additional folding errors, thereby contributing to cell survival. When the stress condition is no longer present, several feedback mechanisms terminate the UPR [16, 17]. However, prolonged ER stress can trigger apoptosis or autophagy.

**Fig. 1:**
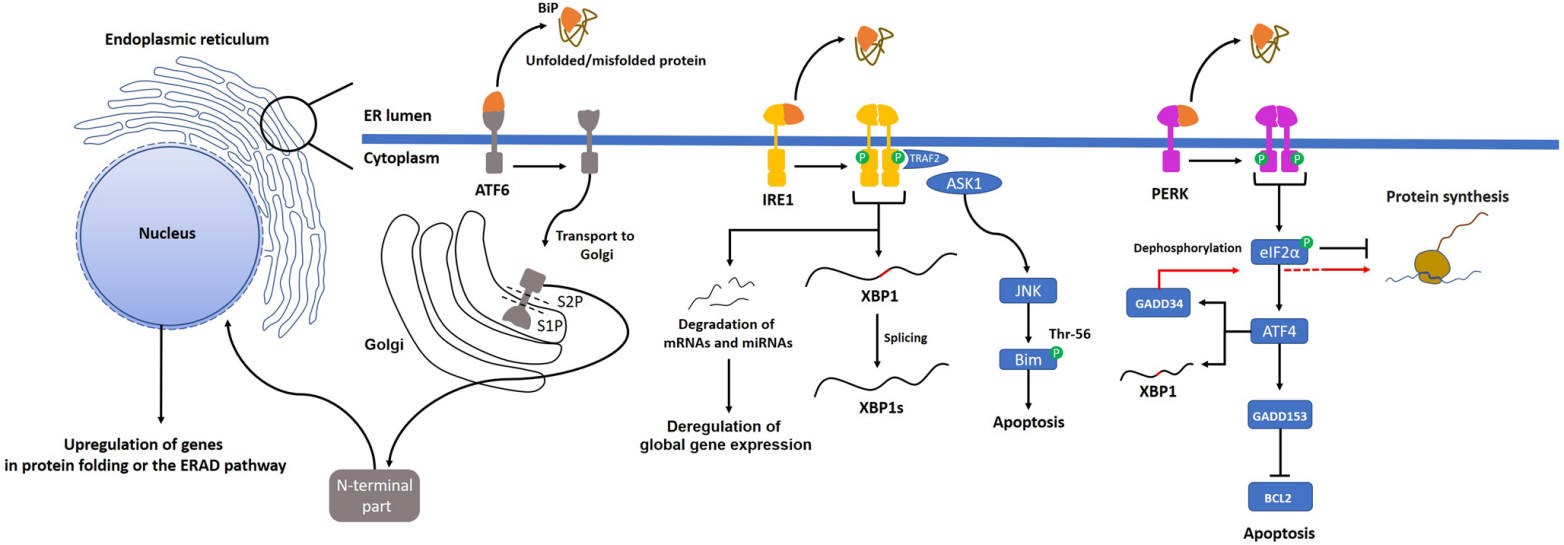
Activation of the UPR mechanism. Accumulation of misfolded and unfolded proteins within the ER is a critical trigger for UPR activation. BiP regulates the function of UPR sensors. In the absence of misfolded proteins, BiP remains bound to the UPR sensor proteins. However, in response to their accumulation in the ER lumen, BiP dissociates from the sensor proteins and binds to these substrates as a molecular chaperone. This activates sensor proteins on the ER membrane. ATF6 is transported to the Golgi apparatus, where it is cleaved by S1P or S2P, resulting in the release of its N-terminal part into the cytoplasm. The cleaved N-terminal portion translocates into the nucleus and acts as a transcription factor, enabling the expression of various genes involved in protein folding and the ERAD signaling pathway. In contrast, dissociation of BiP from IRE1 or PERK results in the homodimerization of each sensor protein. The cytoplasmic region of IRE1 contains both endoribonuclease and serine/threonine kinase domains. Phosphorylation of the protein kinase domains causes two critical events. One of them is the phosphorylation of JNK through TRAF2, which triggers apoptosis. The other is the conformational change of the endoribonuclease domain. Thus, mRNAs are degraded in the cytoplasm, preventing excessive protein synthesis. Endoribonuclease activity also mediates XBP1 splicing. This unconventional splicing of the *XBP1* transcript generates the functional transcription factor XBP1s despite introducing a frameshift. This transcription factor induces the expression of genes involved in protein folding. Following UPR activation, the cells can survive if global protein expression is reduced and misfolded proteins are correctly refolded. PERK activation leads to phosphorylation of eIF2α, which mediates the binding of tRNA_i_^Met^ to the ribosome in a GTP-dependent manner. This situation typically terminates protein synthesis at the initial stage because the phosphorylation of eIF2α prevents GDP-GTP exchange. Although overall protein expression decreases, activating this sensor induces ATF4 expression. ATF4 promotes the expression of *GADD153*, *GADD34*, and *XBP1*, thereby promoting apoptosis, dephosphorylation of eIF2α, and protein folding, respectively.

Inositol-requiring enzyme 1 (IRE1), protein kinase RNA-like endoplasmic reticulum kinase (PERK), and activating transcription factor 6 (ATF6) are involved in the activation of the UPR mechanism (Fig. 1). The function of all three sensor proteins is regulated by GRP78 (also known as BiP), a chaperone protein from the HSP70 family [18]. Like other HSP70 family chaperones, BiP contains a nucleotide-binding domain (NBD) and a substrate-binding domain (SBD) [19]. The binding of ATP to the NBD induces a conformational change in the SBD. ATP hydrolysis promotes substrate binding, and the release of ADP allows the binding of a new ATP molecule to NBD. In the absence of ER stress, BiP binds to sensor proteins on the ER membrane and maintains them in an inactive state [20–22]. However, the binding affinity of BiP to unfolded proteins is higher than that of sensor proteins [23]. Therefore, in the presence of misfolded proteins in the ER lumen, BiP dissociates from the sensor proteins and binds to these structures [24]. This results in the oligomerization of the sensor proteins IRE1 and PERK, or the translocation of ATF6 to the Golgi apparatus. These events are required for the UPR activation.

IRE1, a type I transmembrane protein, includes protein kinase and endoribonuclease domains [25]. Its oligomerization leads to autophosphorylation of the serine/threonine protein kinase domains within the homodimeric structure and activation of the endoribonuclease domain [26]. The conformational activation of the endoribonuclease domain occurs through autophosphorylation. The endoribonuclease domain splices out a 26-bp intron from the mRNA encoding the transcription factor X-box binding protein 1 (XBP1) [27, 28]. Splicing of XBP1 mRNA results in a frameshift that produces a stable and active transcription factor (XBP1s) [28]. This transcription factor then translocates into the nucleus to induce the expression of target genes involved in protein folding and degradation. Induced expression of *XBP1* in HeLa cells significantly increases the expression levels of genes such as A*NK2*, *DNAJB9*, *EDEM1*, *ERLEC1*, and *SEC23B* [29]. Interestingly, high XBP1s expression in these cells enhances the expression of genes that enhance ER folding capacity and attenuate proteotoxic stress, while diminishing the expression of specific stress sensors and apoptosis-related genes [29]. To ensure cellular homeostasis and promote survival, the cell strategically prioritizes protein refolding in the ER to minimize the detrimental effects of misfolded proteins. IRE1 activation also induces mRNA and microRNA degradation, reducing overall protein synthesis in the cell [15, 30]. Following IRE1 activation, protein kinase domains phosphorylate the adaptor protein TRAF2, thereby triggering JNK (c-Jun N-terminal protein kinase) activation via ASK1 [31]. Activated JNK phosphorylates the BH3-only protein Bim at the Thr-56 residue, effectively inducing apoptosis [32]. BH3-only mediators can also activate reticular Bak to initiate an ER-to-mitochondria signaling cascade, leading to cytochrome c release and subsequent apoptosis, independent of the canonical Bax/Bak-dependent mechanism [33]. In this situation, pro-apoptotic proteins are activated. IRE1 responds to ER stress by modulating processes including mRNA degradation, protein folding/degradation, and apoptosis. Beyond these functions, IRE1α signaling regulates oxidative stress, thereby supporting cell survival and responses to DNA damage [13]. Through its downstream effector XBP1s, IRE1 also indirectly modulates various transcriptional programs. XBP1s regulates transcription factors such as AP-1, ATF6, HIF1α, and estrogen receptors, forming physical regulatory complexes that control gene expression associated with differentiation and development. In parallel, the IRE1β isoform has been reported to repress translation by degrading 28S rRNA, affecting ribosome assembly and protein synthesis [13].

Similar to IRE1 activation, oligomerization of PERK results in autophosphorylation of the cytoplasmic kinase domains at the C-terminus [34, 35]. Activated PERK phosphorylates the eukaryotic initiation factor 2α (eIF2α), which plays a crucial role in initiating protein synthesis [35]. In its GTP-bound form, eIF2 delivers the methionine-charged initiator tRNA to the 40S ribosomal subunit [36]. Following the recruitment of mRNA to the ribosomal subunit by eIF4 factors, the complex begins scanning the mRNA for the AUG start codon. Once the codon is recognized, the hydrolysis reaction of eIF2-GTP is catalyzed by eIF5. Subsequently, eIF2 dissociates from the structure, and the 60S subunit joins to this initiation complex. eIF2, through its GTPase activity, plays a crucial role in recognizing the start codon in protein synthesis. Under several stress conditions, phosphorylation of eIF2α inhibits the GDP-GTP exchange, thereby blocking protein synthesis at the initial step [37]. Therefore, PERK activation suppresses global protein synthesis. In addition, PERK activation induces the expression of activating transcription factor 4 (ATF4). ATF4 regulates the expression of *XBP1*, *GADD34* (protein phosphatase-1 regulatory subunit 15A), *GADD153* (also known as CHOP), and various apoptotic genes [17, 38–40]. GADD34 is responsible for the dephosphorylation of phosphorylated eIF2α [17], while GADD153 suppresses the expression of anti-apoptotic Bcl-2 protein [41]. Furthermore, ATF4 is a pivotal mediator associated with autophagy induction by promoting LC3 expression [42]. Bassot et al. have revealed a metabolic adaptation mechanism based on the interaction between PERK and ERO1α (ER oxidoreductin 1) in response to ER stress [43]. PERK localizes to mitochondria-ER interaction sites via ERO1α during the early stage of ER stress. This interaction leads to oxidation and oligomerization of PERK. ERO1α-mediated activation of PERK initiates a stress response distinct from the classical UPR pathway. The PERK-ERO1α complex oxidizes Ca^2+^ transport proteins, such as SERCA2b and IP3R1, thereby enhancing Ca^2+^ transport from the ER to mitochondria. Induced transfer of Ca^2+^ stimulates the Krebs cycle and triggers oxidative phosphorylation in mitochondria, leading to greater ATP production. Thus, PERK and ERO1α maintain cellular energy balance during early ER stress by modulating Ca^2+^ transfer at ER-mitochondrial interaction sites [43]. In addition, PERK signaling through eIF2α phosphorylation promotes the selective translation of ATF4, which regulates genes involved in amino acid import, glutathione biosynthesis, and cellular antioxidant responses [44]. Furthermore, PERK can directly phosphorylate nuclear factor erythroid 2-related factor 2, thereby activating antioxidant pathways that contribute to redox homeostasis under stress conditions [45, 46].

ATF6, a type II transmembrane protein, is another sensor protein in the UPR mechanism. The C-terminal end of ATF6 extends into the ER lumen, while its N-terminal end faces the cytosol. The binding of BiP to the misfolded proteins in the ER results in the transfer of ATF6 to the Golgi apparatus via COPII vesicles [47]. During ER stress, the reduction of disulfide bonds within ATF6 promotes its monomerization and facilitates its activation [48]. In addition, the dissociation of BiP exposes Golgi localization sequences within the luminal domain of ATF6, enabling its trafficking to the Golgi apparatus [48]. Here, ATF6 is sequentially cleaved by the site-1 and site-2 proteases, releasing its N-terminal fragment, ATF6(N), which contains the bZIP DNA-binding domain, into the cytosol [49]. This fragment translocates to the nucleus and induces the transcription of genes involved in protein folding and quality control, including chaperones such as GRP78/BiP and GRP94, as well as transcription factor CHOP [50]. In addition, it also upregulates protein disulfide isomerases (PDIs) and components of the ERAD pathway [50, 51]. ATF6 enhances ER protein-folding capacity, promotes the degradation of misfolded proteins, and contributes to cellular adaptation to ER stress.

UPR activation triggers various signaling pathways that coordinate an appropriate cellular response to ER stress [52]. The level of protein damage within a cell determines whether proteostasis-regulating mechanisms are activated or whether the cell undergoes apoptosis or autophagy. In this context, UPR is interconnected with the NF-κB, Akt, and JNK signaling pathways, all of which coordinate a wide range of cellular processes [52]. These signaling pathways are engaged downstream of UPR activation and coordinate the cellular response to ER stress, rather than acting as independent regulators of proteostasis. Thus, this review specifically focuses on the role of UPR sensors in GBM progression.

### Glioblastoma

**Fig. 2:**
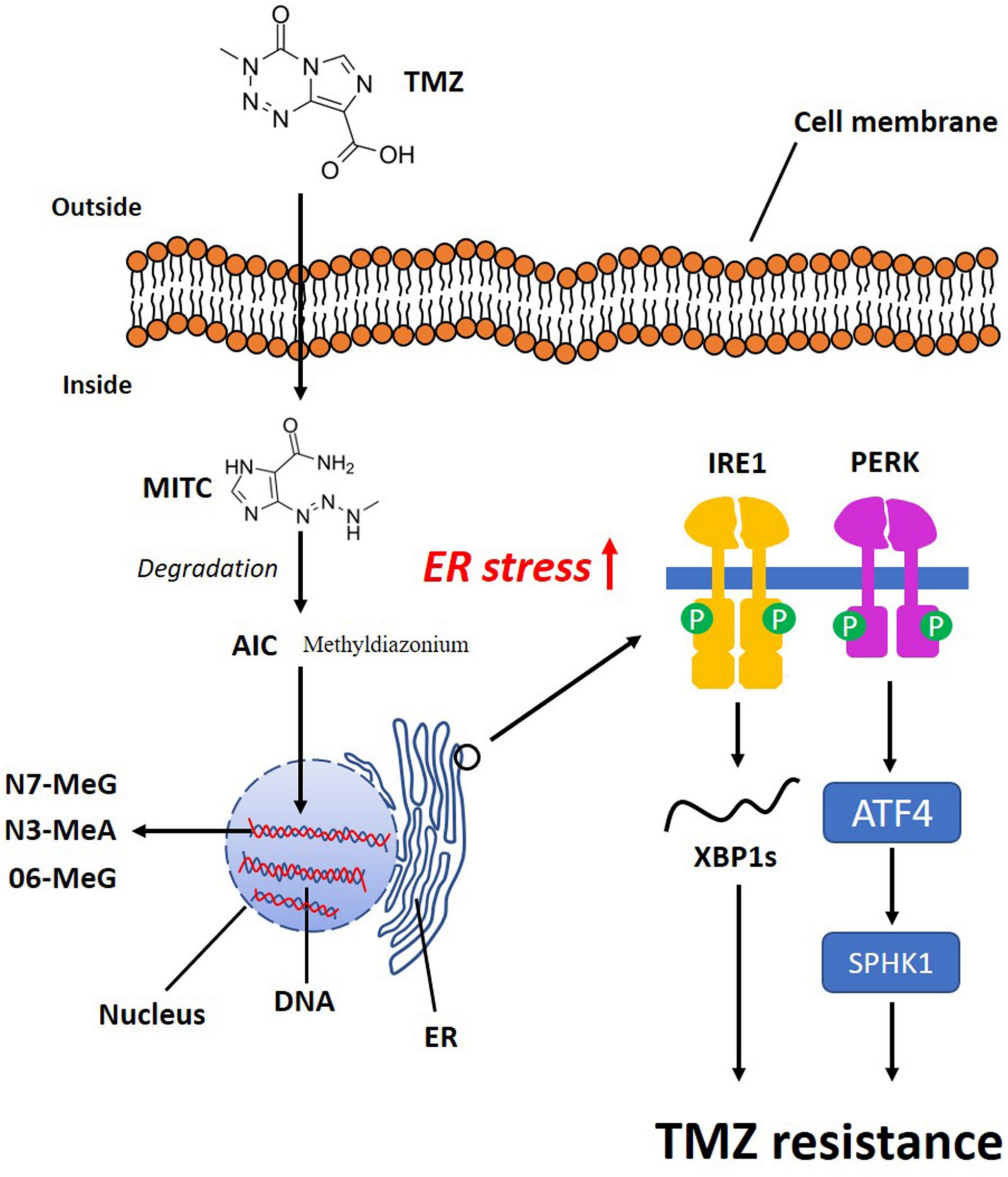
Mechanism of action of TMZ. TMZ undergoes pH-dependent degradation to form MTIC, which subsequently generates a methyldiazonium ion and AIC. TMZ induces DNA damage by transferring methyl groups to guanine and adenine bases. Additionally, it induces ER stress in GBM cells. TMZ-induced ER stress contributes to the development of resistance to TMZ in GBM, primarily through activation of IRE1 and PERK.

GBM is a highly lethal primary brain tumor in adults. The current treatment of GBM consists of surgical resection followed by radiotherapy administered concomitantly with temozolomide (TMZ) [53]. Despite this aggressive multimodal approach, the median overall survival for patients with GBM remains approximately 12–15 months [53]. TMZ is the standard chemotherapeutic agent for treating GBM. TMZ is a lipophilic alkylating agent belonging to the imidazotetrazine class [54]. At physiological pH, TMZ is converted to the active form, MTIC [55]. The chemical degradation increases with increasing pH, leading to the conversion of MTIC to AIC and a methyldiazonium ion [56]. TMZ methylates DNA at the O6 and N7 positions of guanine and the N3 position of adenine, forming DNA adducts [55]. These adducts interfere with DNA replication and transcription, leading to cell cycle arrest and apoptosis. The genotoxic effect of TMZ is mostly associated with O6-methylguanine lesions [55]. Beyond its genotoxic properties, TMZ also induces ER stress, which in turn activates the UPR signaling pathway (Fig. 2). As detailed in the following sections, PERK and IRE1 signaling contribute to UPR activation in TMZ-treated GBM cells [9, 57].

Resistance to TMZ is a major factor limiting therapeutic success. Consequently, improving current treatment strategies for patients with GBM remains an important and actively investigated area of research [55]. Although TMZ is an effective cytotoxic agent, resistance frequently develops in GBM owing to tumor-associated genetic alterations that adversely affect treatment response [58]. Genetic alterations have been identified in GBM and are associated with therapeutic resistance and prognosis. Mutations or abnormal expression of genes such as *IDH1/2*, *MGMT*, *TP53*, *EGFR*, and *PTEN* have been reported to affect GBM progression and treatment response [59]. In addition to molecular heterogeneity, ER stress, hypoxia, and oxidative stress have been implicated in GBM pathogenesis and tumor progression [60–62]. These factors promote the intracellular accumulation of misfolded proteins, initially engaging HSPs and subsequently activating the UPR. Under such conditions, UPR signaling supports adaptive responses that enhance GBM cell survival. As previously described, dissociation of BiP from sensor proteins initiates UPR signaling. In GBM, BiP expression is frequently elevated and correlates with tumor aggressiveness and poor clinical outcome [63–65]. Increased BiP levels enhance the ability of GBM cells to cope with proteotoxic stress, thereby promoting proliferation and therapy resistance [64, 66]. Mechanistically, BiP supports adaptive signaling pathways and suppresses apoptosis under manageable stress conditions. However, under severe or sustained ER stress, BiP-associated UPR signaling can induce apoptosis [66]. Beyond its canonical ER localization, aberrant redistribution of BiP has been linked to altered signaling dynamics in GBM cells [63, 67]. BiP can relocalize to the cell surface in GBM cell lines and the xenograft model. Immunostaining with the anti-GRP78 N-20 antibody reveals a diffuse plasma membrane distribution of BiP, which colocalizes with the cell surface marker N-cadherin [63]. Treatment of surface-localized BiP with the N-20 antibody reduces cell survival and population growth in GBM cell lines [67]. In U87MG cells, gene silencing or N-20 antibody-mediated ligation of BiP on the cell surface reduces Akt and ERK1/2 phosphorylation, indicating that BiP on the cell surface promotes these signaling pathways [63]. This aberrant redistribution of BiP thereby alters intracellular signaling dynamics in GBM. Functional studies have demonstrated that BiP suppression impairs cell growth and sensitizes GBM cells to therapeutic stress, thereby identifying BiP as a potential therapeutic target [64, 67]. BiP plays a central role in activating UPR. Dysregulation of this protein has been shown to contribute to GBM pathogenesis. However, aberrant UPR activation in GBM extends beyond BiP and also arises from alterations in UPR sensor proteins.

### Aberrant Activation of UPR Sensors in GBM

#### IRE1

##### Kinase and RNase Functions in Therapy

The presence of both kinase and ribonuclease domains in IRE1 highlights its potential as a therapeutic target. IRE1 plays a critical regulatory role in the cellular stress response by splicing XBP1 mRNA to produce an active transcription factor and by mediating mRNA degradation. The RNase function of IRE1 depends on phosphorylation of its kinase domains. Therefore, targeting the ATP-binding sites in this domain inhibits its endoribonuclease activity, contributing to an anticancer effect in the U87 cell line and orthotopic GBM tumors [7]. Z4 inhibits RNase activity in GBM cells by binding to the ATP-binding site of IRE1, thereby suppressing XBP1 splicing and regulated IRE1α-dependent decay (RIDD). Treatment with Z4 reduces the migratory capacity of GBM cells and increases their sensitivity to TMZ. Despite its small molecular size, Z4 cannot cross the blood-brain barrier (BBB). In contrast, its derivative, Z4P, can cross the BBB in the vivo model and effectively suppresses IRE1 activity. When this molecule is administered with TMZ, it prevents tumor recurrence with minimal side effects and increases the treatment efficacy by sensitizing GBM cells to TMZ [7]. The suitability of this region in IRE1 for the design of BBB-permeable small molecules represents a significant advantage for GBM treatment. A preclinical study demonstrates the therapeutic potential of inhibiting the IRE1 pathway in GBM [8]. In the study, the GL261 murine GBM cell line, characterized by elevated IRE1 activity, is utilized in orthotopic models. Following surgical resection, mice are treated with a Stupp-like protocol. In vitro, the IRE1 RNase inhibitor MKC8866 suppresses XBP1 splicing in GL261 cells and enhances TMZ sensitivity. This effect is further potentiated when combined with radiotherapy. Although MKC8866 does not cross the BBB, local administration of this inhibitor via fibrin-collagen implants during surgery increases survival in combination with the Stupp-like protocol. Histological analysis indicates that this combination therapy promotes tumor necrosis and apoptosis while decreasing macrophage and microglial infiltration [8]. Overall, targeting IRE1 may improve the efficacy of GBM treatment.

##### IRE1-Induced Apoptosis

Activated IRE1 can directly induce apoptosis in GBM cells. IRE1 activation increases expression of the CD95/Fas death receptor via XBP1s, which, in turn, drives U87 GBM cells to undergo apoptosis [68]. Activation of inositol triphosphate receptors elevates cytosolic calcium levels in the A172 cell line [69]. This situation activates IRE1, ultimately resulting in the nuclear translocation of the pro-apoptotic factor CHOP. The IRE1-CHOP-ERO1α signaling axis promotes calcium-dependent JNK1 activation by inducing oxidative stress. In contrast, suppression of IRE1 and JNK1 in GBM cells results in evasion of apoptotic cell death [69].

##### Interaction with IGF-1 Signaling

IRE1-induced apoptosis in GBM cells is associated with activation of downstream effectors and other UPR sensors. Thus, IRE1 activation may lead to an opposing outcome in GBM cells. SERCA inhibitors have been shown to induce the UPR response in GBM patient-derived neurosphere cell lines [70]. These cell lines are classified as responsive or non-responsive to this inhibitor. Although expression levels of GRP78 and PERK-associated proteins are similar between these groups, IRE1 and ATF4 are overexpressed in non-responsive cells. Moreover, the expression levels of UPR-related genes correlate with those of *IGFBP3* and *IGFBP5*, components of the IGF-1 signaling pathway. While overexpression of these genes is associated with therapy resistance, the deletion of *IRE1*, *IGFBP3*, or *IGFBP5* sensitizes the U251 cell line to the SERCA inhibitor. Inhibition of the IGF-1R receptor, particularly in GBM cells harboring *IRE1* deletion, significantly increases the cytotoxic response to treatment [70].

##### Pro-Inflammatory Functions

In response to ER stress, IRE1 also regulates the expression of pro-inflammatory cytokines in various cancer types [71]. In GBM, IRE1 signaling regulates the expression of the ubiquitin-conjugating E2 enzyme (UBE2D3) through both XBP1s and RIDD activities, contributing to NF-κB activation, which in turn enhances chemokine production and increases myeloid cell infiltration into the tumor microenvironment [72]. IRE1 activation increases the expression of pro-inflammatory chemokines, including CCL2, CXCL2, IL-6, and IL-8, in GBM tumors [72]. Thus, high IRE1 activity may be associated with a pro-inflammatory tumor microenvironment in GBM.

##### Regulation of Proliferation

Inhibition of IRE1 exerts anticancer effects by suppressing proliferative signaling cascades in the U87 cell line [73]. In this context, IRE1 is a central regulator that interacts with multiple signaling pathways involved in the cellular stress response. Targeting this sensor protein holds significant therapeutic promise by concurrently suppressing other molecular pathways implicated in GBM progression and therapy resistance [72]. Epiregulin (EREG), a ligand of the EGFR receptor, is highly expressed in GBM cells and secreted extracellularly [74]. Inhibition of EGFR or EREG with specific antibodies attenuates GBM cell proliferation by blocking the autocrine signaling mechanism. IRE1 inhibition in the U87 cell line and human xenograft tumor models results in a similar decrease in EREG ligand expression. This effect is attributed to the IRE1-mediated activation of JNK [74].

##### Additional Cellular Functions of IRE1

PER1, a target of IRE1-mediated mRNA degradation, regulates the circadian clock in GBM [75]. siRNA-mediated silencing of *IRE1* expression in the U87 cell line prevents PER1 mRNA cleavage. In contrast, *PER1* mRNA expression is reduced in GBM cells overexpressing IRE1. The P-loop structures of *PER1* mRNA are a direct target of IRE1 endonuclease activity. In the in vivo GBM model, silencing *IRE1* increases PER1 protein levels and reduces tumor volume and angiogenesis. Mice bearing PER1 knockdown tumors exhibit reduced overall survival, indicating its role as a tumor suppressor. In addition, low PER1 expression is associated with a poor prognosis in GBM patients [75]. The UPR can be activated in GBM under hypoxic conditions. White et al. identified hypoxia-specific differentially expressed genes (DEGs) in the T98G cell line to screen potential drug targets [76]. Hypoxia-specific DEGs in GBM are associated with glycolysis, hypoxic response, cell adhesion, and, in particular, the IRE1-mediated UPR [76]. IRE1 regulates adaptation to hypoxic conditions in U87 GBM cells by modulating the expression of estrogen-related genes [77]. Under hypoxic conditions, *ESRRA* and *NRIP1* are downregulated, whereas *E2IG5* and *PGRMC2* are upregulated. In GBM cells, simultaneous inhibition of both the kinase and RNase activities of IRE1 results in a similar expression profile for these genes. However, this regulatory effect is impaired under both conditions. Upon IRE1 suppression, *NRIP1* expression is completely abolished under hypoxic conditions, whereas *ESRRA* expression becomes more sensitive to hypoxia [77]. These findings suggest that IRE1 modulates hypoxia-driven gene expression to regulate GBM cell adaptation under hypoxic conditions.

#### PERK

##### PERK Activation and Cell Death

The proliferation of GBM cells is suppressed as a consequence of ER stress-induced cell death [78]. ER stress increases CHOP level and caspase-3 activity in U373 and A172 GBM cells [78]. ER stress activates PERK, which in turn mediates apoptosis and cell cycle arrest through the ATF4-ATF3-CHOP axis in GBM [79]. Surprisingly, PERK activation may not consistently induce apoptotic signals in GBM cells. PERK activation leads to ATF4-mediated transcriptional regulation of downstream target genes. POLR2J is a subunit of RNA polymerase II. Interaction of this subunit with ATF4 promotes transcriptional activation [80]. In GBM cell lines with suppressed *POLR2J* expression, the protein levels of EGFR, p-Akt, and c-Myc decrease, leading to cell cycle arrest at the G0/G1 phase [81]. Consequently, GBM cell proliferation is reduced, and EMT is inhibited, leading to decreased migration and colony-formation capacity. Interestingly, *POLR2J* silencing does not inactivate the UPR in U251 and A172 GBM cells. Instead, eIF2α phosphorylation is significantly elevated, suggesting that ER stress signaling remains active [81]. At this point, treatment with an HDAC inhibitor further enhances PARP-mediated apoptotic signal in GBM cells [81]. These findings indicate that ATF4 downstream pathways also modulate oncogenic processes in GBM.

##### PERK and TMZ Response

**Fig. 3:**
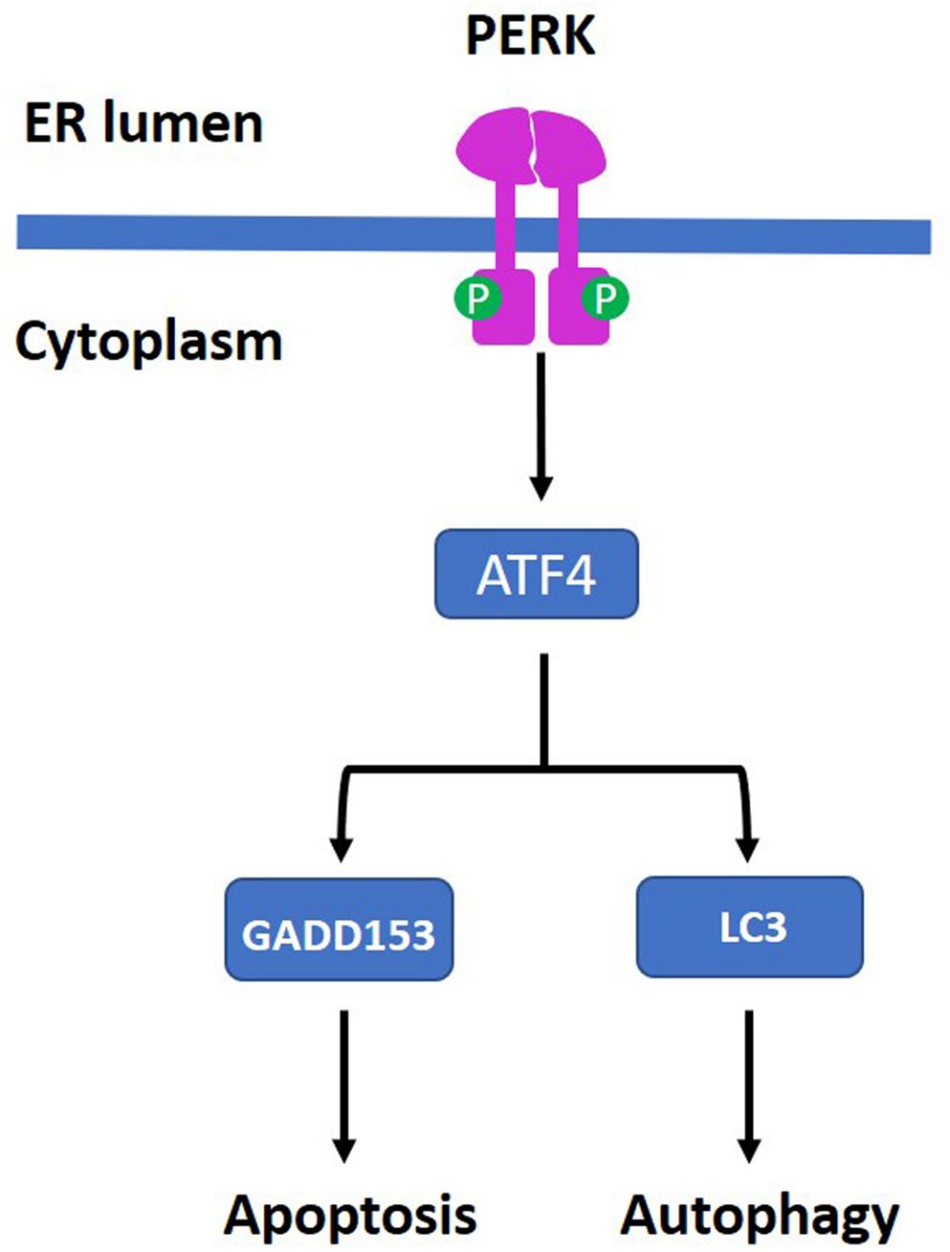
PERK regulates the switch between autophagy and apoptosis. PERK activation induces GADD153 expression via ATF4, thereby triggering apoptosis. In contrast, ATF4 directly promotes LC3 expression, driving cells toward autophagy. Shifting the balance from autophagy to apoptosis in GBM cells may improve the effectiveness of TMZ treatment.

PERK activation is a critical determinant of TMZ efficacy in GBM cells. In TMZ-treated GBM cells, the PERK/eIF2α/ATF4 signaling axis is significantly upregulated, and ATF4-mediated SPHK1 expression contributes to TMZ resistance [57]. Conversely, sustained activation of this signaling route changes how GBM cells respond to TMZ. Upon prolonged ER stress in GBM cell lines, TMZ induces BiP, ATF4, and CHOP expression, resulting in sustained UPR activation [82]. TMZ triggers apoptosis in GBM cells by causing DNA damage. However, DNA repair mechanisms reverse TMZ-induced DNA damage [83]. DNA repair is impaired in GBM cells because ER stress-induced UPR activation reduces RAD51 expression [82]. In this case, loss of DNA repair function and UPR-mediated upregulation of pro-apoptotic genes are key factors that enhance the effectiveness of TMZ. The combination of simvastatin and TMZ has been shown to induce UPR-associated events in GBM cells [9]. This combined treatment increases GRP78 expression, promotes XBP1 mRNA splicing, and enhances eIF2α phosphorylation. Notably, the PERK inhibitor GSK2606414 significantly induces cytotoxicity in GBM cell lines. When combined with simvastatin and TMZ, PERK inhibition further reduces cell viability in the U87 cell line, whereas in the U251 cell line, it shows no substantial additional effect. Instead, PERK inhibition differentially modulated autophagic flux across cell lines, suggesting that the observed cytotoxicity may be associated with an autophagy-dependent mechanism [9]. The autophagy-apoptosis switch following UPR activation may determine TMZ responsiveness in GBM (Fig. 3).

##### PERK and Autophagy

The accumulation of ROS induced by ER stress triggers autophagy in GBM cells via UPR activation [84]. Autophagy maintains cellular homeostasis by eliminating misfolded or aggregated proteins and damaged organelles via lysosomal degradation, thereby promoting cell survival [85]. The activation of autophagy via UPR prevents apoptosis, contributing to chemotherapy resistance in GBM [86]. However, if autophagy degrades cytoplasm excessively, it can lead to autophagic cell death (ACD), underscoring its potential dual role in cell survival and destruction. The autophagy inducer Loperamide (LOP) increases the expression of the ATF4 transcription factor in GBM cell line MZ-54 [87]. In the mechanism of action, this agent induces reticulophagy in GBM cells, a process characterized by the targeting of expanded ER fragments by autolysosomes and lysosomes. This situation triggers ACD in GBM cells. Suppression of ATF4 expression abolishes the LOP-induced cellular effect in GBM cells [87]. The UPR-mediated autophagy blocks apoptosis in GBM [86]. Inhibition of UPR-induced autophagy may potentiate the effect of TMZ on GBM cells. Conversely, if the UPR induces autophagy, this may reduce the effectiveness of TMZ and even lead to resistance.

##### PERK in the Adaptation of GSCs to the ECM

Another important feature of the PERK sensor is its ability to enable GBM stem-like cells (GSCs) to adapt to evolving ECM conditions. When GSCs are cultured in an alginate hydrogel supplemented with human blood plasma, they differentiate into highly proliferative cells with elongated morphology, adapting to their environment [88]. This adaptation takes place through changes in the organization of the F-actin cytoskeleton in GSCs. Interestingly, PERK inhibition decreases F-actin expression, whereas the intracellular cytoskeleton remains unchanged. Additionally, PERK interacts with filamin-A, another cytoskeletal protein, contributing to the adaptive response to mechanical stress [88]. In these cells, PERK also regulates the expression of ECM proteins [89]. In GSCs with suppressed PERK, the expression levels of vinculin and tensin proteins are significantly lower than those in controls, accompanied by a decrease in vimentin expression and an increase in tubulin expression [89]. In this context, the UPR is activated in GSCs under mechanical stress, reorganizing intracellular and extracellular cytoskeletal structures and enabling cells to adapt to changing microenvironmental conditions.

#### ATF6

##### Role in Response to ER Stress

High-resolution proteomic analyses identify the expression levels of key UPR-regulating receptors (PERK, ATF6, IRE1) and effector proteins (ATF4, CHOP, GADD34, XBP1) in GBM and provide comprehensive insights into how their expression profiles change dynamically over time in response to ER stress [90]. Notably, ATF6 exhibits high basal expression, whereas PERK and IRE1 are significantly activated under stress. Furthermore, among the effector proteins, ATF4 and CHOP appear to provide rapid and delayed responses [90]. UPR is not only a response to acute stress in GBM cells but also a key mechanism involved in adapting to prolonged stress conditions. ER stress induces apoptosis via caspase 3/7 by activating the UPR in GSC-rich neurospheres [91]. However, the differentiation of GSCs within neurospheres strengthens their adaptive capacity against ER stress. ATF6 is expressed in neurospheres derived from GSCs, and its expression increases in response to ER stress. Interestingly, suppressing ATF6 in neurospheres does not alter their sensitivity to ER stress [91]. Consistently, another study demonstrated that the induction of ER stress in U87 and U251 cell lines increases the expression of effector proteins, such as p-eIF2α and XBP1s, as well as GRP78 and ATF6 [92]. In this case, apoptosis in GBM cells following UPR activation is driven by ATF4-induced CHOP expression, independent of ATF6 function [92]. Collectively, ATF6 may enhance the adaptive capacity and survival of GBM cells under ER stress conditions.

##### Therapeutic Modulation of ATF6

During the UPR, cell survival under stress conditions relies on ATF6-mediated induction of chaperone proteins and PDIs, which are essential for protein folding. However, when this process fails, expression of pro-apoptotic genes increases. In this regard, betulinic acid (BA) emerges as an effective compound that redirects the UPR from an adaptive to an apoptotic state [93]. BA inhibits the proliferation of both TMZ-resistant and -sensitive GBM cell lines. It also exhibits a synergistic effect with TMZ in GBM. This effect of BA is partially related to the suppression of SP1 (specificity protein 1) expression, which plays a critical role in ATF6-mediated signaling. Under ER stress, ATF6 is transported to the Golgi apparatus, leading to the expression of adaptive proteins, including HSPs and PDIs [50]. However, BA-induced SP1 downregulation prevents initiation of the ATF6-mediated response. As a result, CHOP is induced by PERK activation in GBM cells, leading to apoptosis [93]. Hence, silencing ATF6 may decisively drive GBM cells toward apoptosis rather than survival.

##### Modulation of UPR in GBM by Natural and Synthetic Compounds

Several natural and synthetic compounds have been reported to modulate UPR signaling in GBM. These substances can modulate cell survival, apoptosis, and response to therapy. Table 1 summarizes representative compounds, their mechanism of action, and potential antiglioma effects, highlighting the therapeutic relevance of targeting UPR in GBM. Tunicamycin (TM) is a nucleoside antibiotic produced by various *Streptomyces* species [94]. TM, an N-linked glycosylation inhibitor, induces ER stress and demonstrates pro-apoptotic, anti-proliferative, and anti-invasive effects in different experimental models using GBM cells [78, 95, 96]. The induction of CHOP-mediated apoptosis in TM-treated GBM cells is consistently reported across studies [78, 95, 97]. The TM-induced upregulation of CHOP expression, together with increased caspase-3 activation and PARP cleavage in GBM cells, indicates a shift from the adaptive phase of ER stress to an irreversible apoptotic process [78, 95, 97]. In addition, TM reduces the colony-forming capacity of GBM cells [78]. The observation of these effects at even low concentrations (1 µM) indicates the strong anticancer activity of TM in GBM cells [78, 97]. This effect of TM is not limited to differentiated tumor cells. TM also demonstrates a significant inhibitory effect on GSCs [91, 95]. Cancer stem cells represent a small subpopulation within tumors characterized by therapeutic resistance and high tumorigenic capacity [98]. GSCs are widely recognized as major contributors to chemoresistance and recurrence in GBM [98]. GSC-enriched neurospheres exhibit higher UPR activity than non-stem GBM cells [91]. Studies have shown that TM reduces the neurosphere formation and tumorigenic capacities of GSCs [91, 95]. TM reduces the expression of stem cell markers, particularly SOX2, and elevates the expression of pro-apoptotic markers in these cells. In this context, TM-induced ER stress directly restricts the self-renewal capacity of GSCs [91, 95]. This response is mainly organized by the PERK signaling [91].

**Table 1:** Natural and synthetic compounds that modulate the UPR pathway in GBM

Compound	Source (Natural/Synthetic)	Target in UPR Pathway	Mechanism	Potential Anti-GBM Effect	References
Tunicamycin	Natural	PERK	Induces ER stress, blocks N-linked glycosylation	Apoptotic effect, suppression of stem-like features	[78, 95–97]
Salubrinal	Synthetic	PERK/eIF2α	Inhibits eIF2α dephosphorylation	Apoptotic effect, reduction of cell survival, and increase in sensitivity to PARP inhibitors	[9, 102, 103]
Curcumin	Natural	IRE1, ATF6, XBP1, CHOP	Induces ER stress, increases expression of UPR sensors	Anti-proliferative effect, induction of paraptosis	[107]
GSK2606414	Synthetic	PERK	Blocks PERK kinase activity	cytotoxic effect, sensitization to radiotherapy	[9, 105]
Guanabenz	Synthetic	GADD34	N/A	Suppression of autophagy and induction of cell death	[106]
Z4, Z4P	Synthetic	IRE1	Inhibits the ATP-binding site of IRE1	Sensitization to TMZ	[7]
MKC8866	Synthetic	IRE1	Inhibits XBP1 splicing	Apoptotic and anti-proliferative effects, sensitization to irradiation/chemotherapy	[8]
Loperamide	Synthetic	ATF4	Induces ATF4 expression	Autophagic cell death	[87]
Betulinic acid	Natural	PERK	Suppresses the expression of specificity protein 1	Triggering of apoptosis and DNA damage, sensitization of resistant GBM cells to TMZ	[93]

* N/A* not available

In GBM cells under TM-induced ER stress, inhibition of anti-apoptotic Bcl-2 family members disrupts autophagic cargo degradation and promotes apoptosis [97]. In GBM cells treated with a combination of the pan-Bcl-2 family inhibitor obatoclax (OB) and TM, the stress response is enhanced by upregulation of ATF-4 and CHOP. This leads to apoptotic induction, evidenced by increased caspase-3/7 activity and PARP cleavage. Regarding the UPR, TM specifically activates PERK in GBM cells. Autophagy is induced following LC3-II accumulation and p62 degradation in GBM cells treated with TM alone. Interestingly, combination treatment directs GBM cells toward apoptosis rather than autophagy [97]. In this context, targeting the UPR-mediated adaptive mechanisms may be a critical strategy to improve therapeutic efficacy in GBM. In addition, TM also modulates glycosylation-dependent signaling pathways in GBM [96, 99]. TM-mediated inhibition of N-linked glycosylation in GSCs disrupts EGFR receptor function, thereby suppressing ERK1/2 signaling [96]. This suppression consequently results in markedly reduced cell migration, adhesion, and invasion in GBM cells [96].

The synthetic inhibitor salubrinal selectively inhibits the dephosphorylation of eIF2α [100]. This inhibitor promotes apoptosis and inhibits proliferation in GBM [9, 101–103]. KDELC2 upregulation induces ER stress in GBM, triggers UPR activation, and drives angiogenic cell phenotype [101]. In this process, the PERK-eIF2α-ATF4-CHOP axis plays a central role. Inhibition of this pathway with the PERK inhibitor GSK2606414 reduces angiogenesis and endothelial proliferation, whereas salubrinal maintains eIF2α phosphorylation, thereby sustaining the angiogenic effect [101]. Therefore, ER stress may trigger an adaptive UPR response that promotes vascularization in GBM. Compared to single treatment, salubrinal is more effective in combination therapies in GBM cells. In pediatric GBM (PED-GBM) cells, combined treatment with the histone deacetylase inhibitor vorinostat and PARP1 inhibitors increases eIF2α phosphorylation, thereby reducing cell survival [102]. The significant decrease in cell survival is associated with increased eIF2α phosphorylation in the combination therapy. At this point, the administration of salubrinal or raphin-1, eIF2α dephosphorylation inhibitors, further enhances this effect. Elevated eIF2α phosphorylation increases the sensitivity of PED-GBM cells to PARP inhibitors, thereby enhancing DNA damage-induced cell death. Furthermore, the reduction in eIF2B catalytic subunit expression by these compounds supports their anticancer effects through translational control mechanisms [102]. The combination of simvastatin and TMZ induces apoptosis in GBM cells by inhibiting autophagic flux following UPR activation [9]. While the addition of salubrinal to this combination further elevates eIF2α phosphorylation, it does not lead to a corresponding increase in cytotoxicity. However, treatment with salubrinal alone induces substantial apoptosis in GBM cells, resulting in pronounced cytotoxicity [9]. The combination of salubrinal and TMZ, on the other hand, exerts a synergistic effect in GBM [103]. Salubrinal modulates the transcription of pro-apoptotic genes via the eIF2α-ATF4-CHOP signaling axis in GBM cells. Specifically, the induction of CHOP and the BH3-only protein NOXA plays a critical role in triggering mitochondrial apoptosis. Co-administration of salubrinal with TMZ in GBM cells enhances eIF2α phosphorylation, which in turn reinforces the ATF4/NOXA axis, leading to a significant reduction in cell viability and an elevated apoptotic response [103].

The modulation of the UPR in GBM cells also affects the efficacy of radiotherapy. GSK2606414 is a synthetic inhibitor that targets PERK [104]. Although radiotherapy eliminates most of the treated GBM cells, it induces therapy-induced senescence in the residual cell population [105]. Following radiotherapy, ER stress occurs in the remaining cells, leading to activation of the PERK signaling pathway. This pathway reverses senescence by enabling cells to withstand radiotherapy-induced stress. Treatment of residual senescent GBM cells with the PERK inhibitor GSK2606414 induces apoptosis rather than promoting cell survival [105]. This finding suggests that inhibition of PERK in GBM cells after radiotherapy could prevent tumor recurrence. Other natural and synthetic compounds that modulate UPR signaling in GBM [106, 107] are presented in Table 1.

##### Conclusion and Future Directions

The accumulation of misfolded proteins in the ER triggers the UPR. The GBM microenvironment is characterized by multiple stressors that can promote protein misfolding, necessitating tumor cell adaptation to both acute and sustained ER stress. In this context, UPR appears to support GBM cell survival. By attenuating global protein synthesis while enhancing HSP expression under stress conditions, GBM cells may alleviate proteotoxic stress and maintain cellular homeostasis. Furthermore, differential activation of UPR branches is associated with distinct cellular outcomes, including apoptosis, autophagy, and adaptive survival, in GBM. Studies suggest that targeting UPR sensors can shift this adaptive balance toward apoptosis in GBM. In particular, suppression of UPR sensors, which modulates both autophagy and adaptive response under ER stress, may improve therapeutic responses in GBM, including increased sensitivity to TMZ. In this context, selective inhibition of UPR sensors may be required to effectively eliminate adaptive responses in GBM. Future studies should identify the specific roles of UPR sensors in distinct GBM cell populations, such as GSCs, and develop combined treatment strategies to target UPR-driven adaptive and pro-survival responses in GBM.

## Data Availability

Data sharing is not applicable to this article as no datasets were generated or analyzed.
